# BRCA1 tumor suppressor network: focusing on its tail

**DOI:** 10.1186/2045-3701-2-6

**Published:** 2012-02-27

**Authors:** Bin Wang

**Affiliations:** 1 Department of Genetics, The University of Texas M.D. Anderson Cancer Center, 1515 Holcombe Blvd, Unit 1010, Houston, TX 77030, USA; 2 Genes and Development Program, The University of Texas Graduate School of Biomedical Sciences, Houston, TX 77030, USA

## Abstract

Germline mutations of the *BRCA1 *tumor suppressor gene are a major cause of familial breast and ovarian cancer. BRCA1 plays critical roles in the DNA damage response that regulates activities of multiple repair and checkpoint pathways for maintaining genome stability. The BRCT domains of BRCA1 constitute a phospho-peptide binding domain recognizing a phospho-SPxF motif (S, serine; P, proline; × varies; F, phenylalanine). The BRCT domains are frequently targeted by clinically important mutations and most of these mutations disrupt the binding surface of the BRCT domains to phosphorylated peptides. The BRCT domain and its capability to bind phosphorylated protein is required for the tumor suppressor function of BRCA1. Through its BRCT phospho-binding ability BRCA1 forms at least three mutually exclusive complexes by binding to phosphorylated proteins Abraxas, Bach1 and CTIP. The A, B and C complexes, at lease partially undertake BRCA1's role in mechanisms of cell cycle checkpoint and DNA repair that maintain genome stability, thus may play important roles in BRCA1's tumor suppressor function.

## 

Germline mutations of the *BRCA1 *tumor suppressor gene are a major cause of familial breast and ovarian cancer [1,2]. BRCA1 plays critical roles in a number of diverse cellular processes that ensure genome integrity and the increase risk of breast and ovarian cancer caused by mutation of *BRCA1 *has been attributed to increased genomic instability. To safeguard genome, cells have evolved a defensive mechanism, called the DNA damage response (DDR), to coordinate multiple cellular responses including DNA repair, cell cycle checkpoint regulation, transcription, senescence or apoptosis etc., to counteract genotoxic stress [3-6]. BRCA1 appears to act as a central mediator of the cellular response to DNA damage that regulates the activities of multiple repair and checkpoint pathways [3,5,7-10]. BRCA1 is a substrate of the central DNA damage response kinases ATM/ATR that control the DDR. It is required for homology directed repair, a pathway that facilitates error-free repair of double-strand breaks (DSBs) and resolution of stalled DNA replication forks through homologous recombination (HR) [9-11] as well as postreplicative repair in response to UV damage [12]. Recently it is suggested that much of BRCA1's role in maintaining genome stability is accounted for by its role in maintaining heterochromatin integrity via H2A ubiquitination [13].

BRCA1 associates with multiple repair proteins and cell cycle regulators and such a capability to form multiple protein complexes contributes to its role in maintaining chromosome stability and tumor suppression (Figure 1). BRCA1 is a large protein of 1,863 amino acids. It contains two important domains at each end of the protein, a RING domain at the N-terminus and two BRCT domains at the C-terminus. Many clinically important mutations of *BRCA1 *gene frequently target these two domains. BRCA1 dimerizes with BARD1 through the RING domain present on each or the protein, forming an ubiquitin E3 ligase [14,15]. Earlier studies suggested that the E3 ligase activity of BRCA1 is essential for the DDR and tumor suppression function of BRCA1 [16-19]. Although a recent study using mouse embryonic stem cells and knock-in mouse models suggested that the E3 ligase activity of BRCA1 is not required for homology-directed repair of DSBs and tumor suppression [20,21], the exact role of BRCA1 E3 ligase activity in DNA damage induced ubiquitin signaling and tumor suppression remains obscure. Another study examining mice carrying a pathogenic missense mutant of BRCA1 (C61G), which not only inactivates the E3 ligase activity but also disrupts BRCA1 interaction with BARD1 [19], showed that this mutation, although compromised tumor suppression function of BRCA1, affects response to therapy possibly through residual activity of this mutant in DNA repair [22]. On the basis of in vitro assays, a number of ubiquitination substrates have been proposed for BRCA1/BARD1 E3 ligase, including histones, γ-tubulin, CTIP and BRCA1 itself, however, very few have been reported as substrates in vivo [23-25]. It has also been suggested that BRCA1/BARD1 is capable of interacting with various E2s directing either mono-ubiquitination, or polyubiquitination with different linkages, such as lysine 63 (K63)-, lysine 48 (K48)- or lysine 6 (K6)- linkages [26,27]. A recent study suggested that the BRCA1 mediated histone ubiquitination is required for its role in suppressing satellite DNA repeats transcription in the heterchromatin region and maintenance of genome stability [13]. However it is still not clear whether BRCA1's role in maintaining histone H2A ubiquitination at heterochromatin satellite DNA repeats is important for tumorigenesis.

**Figure 1:**
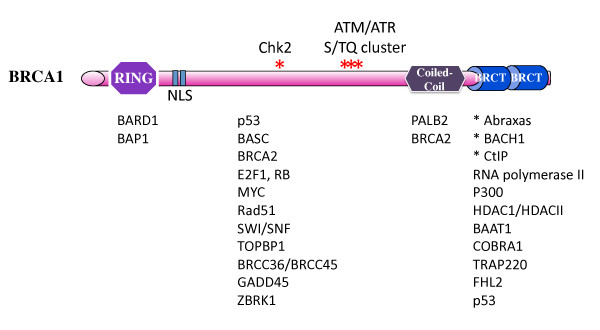
**BRCA1 domains and interacting proteins**. BRCA1 contains a RING domain at its N-terminus, two BRCT domains at the C-terminus and a coiled-coil domain upstream of BRCT domains. The interacting proteins are shown under the region of BRCA1 required for their association. BRCA1 forms an E3 ligase with BARD1 through its RING domain dimerizing with a RING domain containing protein BARD1. A ubiquitin hydrolase BAP1 also interacts with this region. The C-terminal BRCT domains form a phospho-binding module recognizing a phospho-SPxF motif. Abraxas, Bach1 and CtIP have been shown directly bind to BRCT domain through the phospho-SPxF motif. PALB2 binds to the coiled-coil domain of BRCA1 and bridges BRCA1-BRCA2 interaction. A number of other proteins have also been indicated binding to the C-terminal region of BRCA1 or the central region of BRCA1. BRCA1 contains a S/TQ cluster that is phosphorylated by ATM/ATR at multiple sites. In addition, BRCA1 is also a substrate of Chk2

## BRCA1 forms three different complexes through its C-terminus BRCT domains

The BRCT domains of BRCA1 constitute a phospho-peptide binding domain recognizing a phospho-SPxF motif (S, serine; P, proline; × varies; F, phenylalanine) [28-30]. The fact that the BRCT domains are frequently targeted by many clinically important mutations and most of these mutations disrupt the binding surface of the BRCT domains to phosphorylated peptides indicates that they are integral for BRCA1's tumor suppressor function [31]. A recent study of mice carrying a BRCT mutant of BRCA1 that is defective in recognition of phosphorylated proteins and mice carrying an E3 ligase defective mutant of BRCA1 indicates that BRCT phosphoprotein recognition but not the E3 ligase activity is required for BRCA1 tumor suppression [20,21].

**Figure 2:**
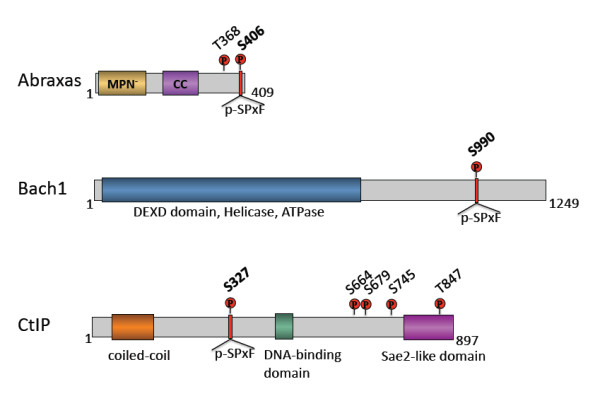
**Three proteins Abraxas, Bach1 and CtIP contain p-SPxF motif that binds to the BRCT domains of BRCA1**. Schematic diagram showing protein domains of three BRCA1-BRCT associated proteins. The phospho-Ser-Pro-X-Phe (p-SPxF) motif is illustrated on each protein. In addition, Abraxas contains a MPN- domain that binds to Ub and a coiled-coil domain that interacts with BRCC36. Abraxas also contains a ATM/ATR phosphorylation site (T368). Bach1 contains a helicase domain. CtIP contains a coiled-coil domain at its N-terminus that is responsible for dimerization of the protein. T847 of CtIP is phosphorylated by CDK and the phosphorylation is required for its ability to promote DNA end resection. CtIP is also phosphorylated by ATM/ATR at S644, S679 and S745. The 509-557 region of CTIP binds to DNA directly in vitro and is required for recruitment to DSB in cells [34]

Although multiple proteins have been suggested to associate with the C-terminus of BRCA1 protein, so far, three proteins Abraxas (also known as Abra1, CCDC98), Bach1 (also known as Brip1, FancJ) and CtIP (also known as RBBP8) have been shown directly interact with the BRCT domains of BRCA1 through the phospho-SPxF motif in a phosphorylation-dependent manner forming mutually exclusive complexes, which were thus named as the A, B and C complexes of BRCA1 [29,32,33] (Figure 2). Although it remains largely unknown how these complexes transmit BRCA1 signaling, it appears that these three complexes of BRCA1 are involved in multiple functions of BRCA1 and thus may be essential for BRCA1's tumor suppressor function. This review will thus focus on a summary of the formation and functions of these three BRCA1-BRCT associated complexes.

## BRCA1-A complex is a deubiquitinating (DUB) complex

The BRCA1 A complex contains at least five different components: Rap80, Abraxas, NBA1 (also known as MERIT40), BRE (also known as BRCC45) and BRCC36 [32,35-41]. Abraxas interacts with the BRCT domains of BRCA1 through a phospho-serine group (p-S406) at the C-terminus of Abraxas, and mediates the interaction of the A complex with BRCA1. In fact, Abraxas appears to serve as a central organizing adaptor protein that not only mediates the interaction of the A complex and BRCA1 but also bridges the interaction of each member of the BRCA1-A complex [32,35,42]. It is worth noting that NBA1 and BRE interaction is critical for maintaining the integrity of the BRCA1-A complex [43]. NBA1 interacts with BRE through a C-terminal conserved PxxR (P, proline; × varies; R, arginine) motif of the NBA1 protein and a C-terminal UEV domain of the BRE protein. Knockdown of NBA1 or BRE leads to decreased levels of components of the complex and compromised BRCA1-A complex function [43].

Modification of proteins by the covalent attachment of ubiquitin (Ub) to the lysine residue of a target protein is a key regulatory mechanism of many cellular processes including DNA damage response [44,45]. Ubiquitination occurs through a three-step process involving Ub-activating enzyme (E1), Ub-conjugating (E2) and Ub-ligating (E3) enzyme. The types of Ub modifications that can form are diverse. Monoubiquitin occurs as a single Ub molecule is attached. Alternatively, a polyubiquitin (polyUb) chain is formed when one of the seven lysine residues side chains within ubiquitin is linked to the C-terminal glycine of another ubiquitin. The best-characterized linkages to date utilize ubiquitin lysine 48 (K48) and lysine 63 (K63). K48-linked polyUb predominantly targets proteins for proteasomal degradation, whereas K63-linked polyUb more often regulates protein function, subcellular localization, or protein-protein interactions. K63 polyUb chain linkage plays important roles in the recruitment of repair factors in the DDR [3].

Protein ubiquitination is a reversible process, and it has become increasingly obvious that Ub deconjugation plays important roles in regulating the Ub-dependent pathways [46-49]. Deubiquitinating enzymes (DUBs) catalyze the removal of Ub from Ub-conjugated substrate proteins. DUBs also function in processing of Ub precursors and Ub adducts. BRCC36 in the BRCA1 A complex is a deubiquitinating enzyme (DUB). BRCC36 was initially identified as a BRCA1 associated protein that contains a MPN^+^/JAMM domain indicative of a metalloprotease activity [50]. It is later found indeed having a deubiquitinating activity specifically toward K63-polyUb linkages [38,51].

One unique feature of the BRCA1 A complex is that multiple ubiquitin binding motifs exist in the BRCA1 A complex. Four members of the BRCA1 A complex, Rap80, Abra1, BRE, and BRCC36, possess polyUb chain binding capability while only the UIM domains of Rap80 display Lys63-linkage specific polyUb binding [35]. BRCC36, Abraxas and BRE, which contains MPN^+^, MPN^- ^and UBC domain respectively, appear to prefer binding to longer polyUb chains with no apparent linkage specificity [35]. Thus, it is likely that the BRCA1 A complex is assembled in a specific manner to facilitate the DUB activity of BRCC36. Interestingly, the BRCA1 A complex bears similarities with the lid of the 19S proteasome regulatory complex [35]. The 19S proteasome lid [52,53] cleaves ubiquitin from substrates and facilitates the entry of substrates into the catalytic proteasome core for degradation [54-56]. In the lid, Rpn11 contains an active MPN^+^/JAMM motif that posesses a DUB activity for substrate deubiquitination, while Rpn8 contains an enzymatically inactive MPN^- ^domain. Similarly, the BRCA1 A complex contains a MPN^+^/MPN^- ^pair present in BRCC36 (MPN^+^) and Abraxas (MPN^-^) proteins. These two proteins dimerizes with each other through a coiled-coil domain on each of the protein. In addition, while a component of the 19S proteasome lid, Rpn10/S5a, contains UIM domains and a VWA domain [57], in the BRCA1-A complex, Rap80 possesses UIM domains at its N-terminus and NBA1 contains a VWA domain. Although the significance of this similarity is not clear, it is likely the BRCA1 A complex is assembled to facilitate the cleavage of ubiquitin from substrates. Indeed, it was shown that interactions of BRCC36 with other components of the complex are critical for the optimum DUB activity of BRCC36 [37,58]. In the BRCA1 A complex, the UIM domains of Rap80 bind specifically to K63-linked polyUb chains while the deubiquitinating enzyme BRCC36 displays an specificity toward these chains [35,38,40,59]. Therefore, the BRCA1 A complex appears to be a DUB complex processing K63-linked polyUb signals during the DDR.

## BRCA1-A complex mediates DNA damage induced ubiquitin signaling for recruitment of BRCA1 to DSBs

Ubiquitination occurs at the double strand breaks (DSBs) in a central DNA damage kinases ATM/ATR dependent manner [3,4,6]. Upon DNA damage, a damage induced ATM/ATR phosphorylation on Ser139 of histone H2AX directly recruits MDC1 through MDC1's BRCT domains. MDC1 itself is a substrate of ATM/ATR, and its phosphorylation leads to the recruitment of an ubiquitin E3 ligase/E2 conjugase, RNF8/UBC13, to damage sites [42,60-62]. Another chromatin-associated ubiquitin E3 ligase RNF168 was also identified working with Ubc13 to amplify the RNF8-dependent polyubiquitin (polyUb) signal that is required for BRCA1 recruitment [63,64]. The RNF8/RNF168 dependent ubiquitination is further facilitated by yet another E3 ligase HERC2 [65,66]. The subsequent ubiquitination events on the damaged chromatin create docking sites for DNA damage repair proteins such as 53BP1 and BRCA1 [3,4,6]. In a parallel pathway, H2AX ubiquitaination by PRC1 also contributes to BRCA1 recruitment [67]. So far, histones H2A and H2AX have been indicated as substrates of these E3 ligases although yet unidentified substrates at DSBs may also likely exist. A recent report indicates that MDC1 is likely to be another substrate with a lysine residue in the MDC BRCT domain ubiquitinated in a Ubc13-dependent manner for interaction and optimum recruitment of Rap80 [68]. Unlike the canonical Lys48-linked polyUb that targets the substrate protein for proteasomal degradation, RNF8/RNF168-dependent ubiquitination around DNA damage sites appears to generate K63-linked polyUb for recruitment of DNA repair proteins [3,60-64].

The BRCA1 A complex accumulates to DSBs in a RNF8/RNF168 dependent manner. The two UIM domains of Rap80 and their capability to bind K63-polyUb are required for accumulation of Rap80 and other components of the BRCA1-A complex to sites of damage [32,38,40,59]. The BRCA1 A complex plays an important role in recruiting BRCA1 to DNA damage sites. Down regulation of each component of this complex compromised the recruitment of BRCA1 to DNA damage sites, leading to increased cell sensitivity to ionizing radiation (IR) and inability of cells to arrest the cell cycle as well as compromised homology recombination-dependent repair in response to DNA damage [32,35,36,38,40,69,70]. The exact role of the BRCA1-A complex in DSB repair and BRCA1 signaling in tumor suppression is still not clear. It is suggested that BRCC36 antagonizes RNF8-Ubc13 dependent ubiquitination such that inhibition of BRCC36 leads to an increased accumulation of RNF8/Ubc13-dependent ubiquitination at DSBs [71]. In addition, it has been suggested that BRCC36 and BRE might be involved in regulating the E3 ligase activity of BRCA1/BARD1 [50]. Nevertheless, the fact that both the BRCA1/BARD1 E3 ligase and the BRCC36 deubiquitinating enzyme are present at DNA damage sites raises the possibility that additional ubiquitin modifications might be necessary to enhance or maintain the ubiquitin signaling events initiated by the RNF8/RNF168 ligases. Functional analyses of the BRCA1-A complex should allow us to further dissect the role of BRCA1 in the DDR and tumor suppression.

## An independent DUB complex formed by Abraxas paralog ABRO1

Abraxas protein has a paralog ABRO1 (Abraxas brother 1), which is 39% identical to Abraxas at the N-terminal region including both a MPN^- ^domain and a coiled-coil domain yet lacking the phospho-SPxF motif at its C-terminus [32,37,43,58,72]. Interestingly, although ABRO1 does not interact with Abraxas, it assembles a DUB complex in a similar manner as Abraxas with several components of the BRCA1 A complex including BRCC36, NBA1 and BRE. While Abraxas mainly localizes in the nucleus mediating the interaction of A complex with BRCA1, ABRO1 is mainly localized in the cytoplasm. Because it lacks the BRCA1 interacting motif, ABRO1 does not interact with BRCA1. The ABRO1 complex (also known as BRISC complex) contains at least ABRO1, BRE, NBA1, and BRCC36. ABRO1 and BRCC36 interaction is required for the DUB activity of BRCC36 in vitro [72]. While Rap80 localizes in the nucleus and is only present in the Abraxas/BRCA1-A complex, BRCC36, BRE, and NBA1 are expressed both in nucleus and cytoplasm and are present in both complexes. The ABRO1/BRISC complex appears to associate with the COP9 signalosome [51]. It was also suggested that ABRO1/BRISC deficiency enhances formation of the BRCA1-Rap80 interaction and increased BRCA1 levels at the DNA damage sites [37,58]. However, the exact role of the ABRO1/BRISC complex needs further investigation to be understood.

## BRCA1-A complex and Cancer

A screen of non-*BRCA1 *or *BRCA2 *mutant familiar breast tumors identified a in-frame deletion of residue 81 glutamic acid of Rap80 that displays reduced ubiquitin binding and compromised ability to recruit A complex and BRCA1 to DSBs [73]. Recently, two genome wide association studies (GWAS) implicated that common genetic variants in NBA1 may predispose women to serous ovarian or hormone negative breast cancer [74,75]. In addition, *BRCC36 *gene is abnormally expressed in several breast cancer cell lines and a subset of sporadic breast tumors [50]. Together, it indicates that BRCA1-A complex may play an important role in breast tumor development.

## BRCA1-B complex is required for replication stress induced checkpoint control and DNA interstrand crosslink repair

The BRCA1-B complex is formed through phosphorylated S990 of Bach1 binding to BRCA1 BRCT [29]. Bach1 Ser990 is phosphorylated by CDK in a cell cycle-dependent manner [29,76]. Earlier, it was found that Bach1 is required for progression through S phase [77] and is required for cell cycle checkpoint that accumulates cells at G2 phase (G2 accumulation) after DNA damage [29,76]. In fact, Bach1 interacts with TopBP1 forming a complex that is required for replication stress induced checkpoint [78]. Both TopBP1 and Bach1 are required for the extension of single stranded DNA regions and RPA loading following replication stress [78].

Bach1 was first identified as a DEAH helicase domain containing protein that binds to the BRCT domains of BRCA1 [79]. It was then discovered to be a member of the Fanconi anemia protein, FancJ [80-83]. Fanconi anemia (FA) is a rare genetic disorder associated with various developmental defects and a high incidence of malignancies [84-86]. The FA patients' cells are extremely sensitive to agents that induce DNA interstrand crosslinks [84-86] indicating that Bach1 plays a role in the DNA interstrand crosslink repair.

In vitro studies suggest that Bach1 is a DNA dependent ATPase, which unwinds DNA in a 5' to 3' direction [87]. Furthermore, Bach1 preferentially binds and unwinds DNA substrates that mimic an intermediate step in homologous recombination [88]. Indeed, Bach1 is required for DNA repair through HR. Expression of a helicase dead mutant of FancJ in cells results in the accumulation of unrepaired DNA breaks and decreased HR [79]. In addition, depletion of Bach1 by siRNAs was also shown to compromise HR [82,86]. Bach1 enzyme activity as well as the BRCA1-FancJ interaction is essential for DNA repair, checkpoint activation and tumor suppression [29,89,90].

Multiple germline mutations that disrupt Bach1 enzyme activity or BRCA1 association have been identified in breast cancer indicating Bach1 is a tumor suppressor [89]. Recently a frame shift mutations in the Bach1 gene that greatly affect the risk of invasive ovarian cancer has also been identified in ovarian cancer [91].

## BRCA1-C complex is required for DNA end resection

The BRCA1-C complex consists of CtIP and the MRN (Mre11/Rad50/Nbs1) complex. BRCA1 forms such a complex with CtIP and MRN in a cell cycle-dependent manner during S and G2 phase of the cell cycle through the BRCT domains of BRCA1 binding to phosphorylated S327, a CDK phosphorylation site of CtIP in a phospho-SPxF motif [76,92]. CtIP is a functional homolog of yeast Sae2 and is required for DNA end resection at the initial step of homologous recombination (HR) dependent DSB repair [93,94]. CtIP promotes DNA end resection by interacting and stimulating the nuclease activity of the MRN complex. The complex formation of BRCA1-CtIP-MRN is important for facilitating DSB resection to generate single-stranded DNA that is needed for HR-mediated DSB repair [92].

Similar to Sae2, of which CDK-dependent phosphorylation promotes resection activity, CDK phosphorylation of CtIP at T847 is required for DSB resection and subsequent HR in the S and G2 phase [95,96]. Recruitment of CtIP to DSB requires MRN [34,92,97]. Both of the N-terminus and C-terminus of CtIP have been indicated to interact with the NBS1 subunit of the MRN [92,94,97]. CtIP recruitment to damage sites also requires ATM kinase activity [34]. It was shown that S664/S745 phosphorylated by ATM is required for CtIP recruitment to DNA damage sites and DSB end resection [98]. It was also suggested that following DNA damage the ubiquitination of CtIP by BRCA1 is critical for the recruitment of CtIP towards a chromatin fraction [25]. In addition, like Sae2, CtIP is acetylated, and deacetylation of CtIP by SIRT6 promotes DNA end resection [99,100].

CtIP is also implicated a role in microhomology-directed alternative nonhomolgous end-joing pathway (alt-NHEJ) and the CtIP-mediated alt-NHEJ has a primary role in chromosome translocation formation and class switch recombination [101,102]. A study in chicken DT40 cells analyzing a mutant of CTIP that fails to associate with BRCA1 suggested that BRCA1-CtIP interaction is required for HR-mediated repair during S-G2 phase progression but involved in microhomology-mediated NHEJ in a BRCA1-independent manner in G1 phase of the cell cycle [103]. Yet another study in chicken DT40 cells suggested that CtIP interaction with BRCA1 is not required for HR-mediated repair [104]. While the discrepancy needs to be further analyzed, the biological significance of the complex formed between BRCA1 and CtIP has not yet been fully studied in mammalian cells.

CtIP is an essential gene in mammalian cells, inactivation of which is embryonic lethal in mice [105]. However, haploid insufficiency of CtIP predisposes mice to multiple types of tumor indicating CtIP is a tumor suppressor [105]. Consistent with its role in the DDR, a recent report showed that mutations of CtIP that generates a dominant-negative C-terminus truncated form of CtIP protein causes Seckel and Jawad Syndromes which is a genome instability disorder and associated with cancer predisposition [106].

## Functions of BRCA1 A, B and C complexes in DNA DSB repair

Eukaryotic cells possess at least three distinct mechanisms for DSB repair including NHEJ (non-homologous end joining), SSA (single-strand annealing) and HR (homologous recombination) [4,107]. NHEJ involves the direct re-ligation of the broken DNA ends and can be error prone if terminal bases are removed prior to ligation. SSA utilizes short stretches of sequence homology flanking the break following DSB resection for annealing resulting in deletion of intervening sequences and loss of genetic information. In contrast to the error-prone repair mechanisms NHEJ and SSA, HR accurately repair DSBs in S-phase and G2-phase cells. HR is initiated by nucleolytic processing of the DSB end to generate 3' ssDNA (single-stranded DNA) overhangs. In the presence of ATP, Rad51 forms a helical nucleoprotein filament on ssDNA that is needed for invasion of the ssDNA into a donor sister chromatid or homologous chromosome to form a joint molecule for homologous recombination. The BRCA1-C complex plays a critical role in the initial resection of DSB end. This is followed by long-range resection dependent on the DNA exonuclease Exo I and the helicase Bloom syndrome protein (BLM), which provides the ssDNA substrates for HR [107]. The BRCA1-B complex also plays a role in HR, yet its specific mechanism remains unclear. BRCA1 associates with BRCA2 through PALB2 [108-110]. BRCA2 promotes the loading of Rad51 to ssDNA for efficient HR repair [111]. Intriguingly it is still not clear how the BRCA1-A complex is involved in HR. Although an earlier report indicated BRCA1 A complex promotes HR since HR is compromised in BRCA1-A complex deficient cells [32], more recent reports suggest that BRCA1-A complex supresses HR such that with depletion of Rap80 or Abraxas, both the B and C complexes assembly at DSB was increased and HR was hyperactivated [112,113]. While it awaits further study for a better understanding of how each of the complex contribute to HR, it is clear that BRCA1 BRCT is critical for HR at least partially through forming the A, B and C complexes.

## Functions of BRCA1 A, B and C complexes in DNA damage induced checkpoint regulation

Studies on A, B and C complexes of BRCA1 indicate that these complexes carry out functions of BRCA1 in cell cycle checkpoint control. Both A and C complexes of BRCA1 have been indicated critical for G2-M checkpoint control in response to IR to ensure that entry into mitosis is transiently inhibited to avoid aberrant chromosome segregation [32,35,36,38,40,69,70]. Interestingly knocking down any components of the A complex such as Rap80, Abraxas, NBA1, BRE, BRCC36 or the C complex component CtIP, the phenotype of G2-M checkpoint deficiency is much milder compared to cells depleted of BRCA1 indicating that only partial function of BRCA1 in G2-M checkpoint control is defective when individual complex was deficient [32,35,36,38,40,69,70,76]. In fact, synthetic knockdown of both Abraxas and CtIP proteins led to a G2-M checkpoint defect comparable to BRCA1-deficient cells [32] indicating that BRCA1 function in G2-M checkpoint control might involve both of the A and C complexes. While the B complex does not appear to be involved in the transient G2-M checkpoint control [76], it is required for G2-accumulation checkpoint that is also observed for BRCA1-deficient cells [29,76,114]. The mechanism of the G2-accumulation checkpoint is still not well defined. In addition, the B complex of BRCA1 is also involved in the replication checkpoint control in response to replication stress [78]. Since BRCA1 appears to be required for multiple checkpoints regulation including the replication checkpoint and G2-M checkpoint control, different BRCA1 associated complexes may only carry out partial of its function in distinctive checkpoints.

## Concluding remarks

**Figure 3:**
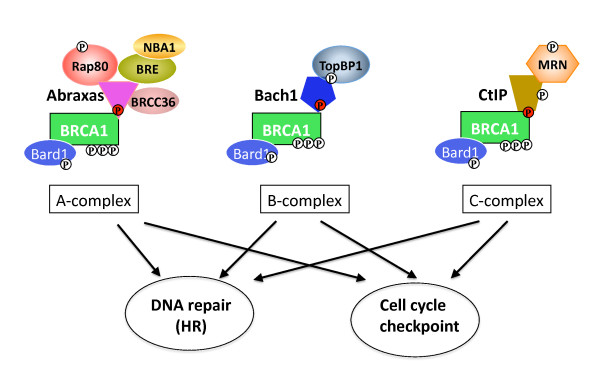
**A, B and C complex of BRCA1 contributes to BRCA1's role in cell cycle checkpoint regulation and DNA repair**. In addition to forming C-terminal associated complexes, BRCA1 contains a coiled-coil domain upstream of BRCT domain, which interacts with a coiled-coil domain at the N-terminus of PALB2. PALB2 associates with BRCA2 thus bridging the interaction of BRCA1 and BRCA2 [117-119]. The N-terminus RING domain, in addition to dimerizing with BARD1 forming an E3 ligase, this region is also reported to interact with a ubiquitin hydrolase BAP1 [120,121]. Previous studies suggested that the central region of BRCA1 is also reported to interact with multiple proteins either directly or indirectly [115,116]. In addition, BRCA1 contains a S/TQ cluster that is phosphorylated by ATM/ATR at multiple sites and the phosphorylation is critical for BRCA1's role in cell cycle checkpoints regulation in the DDR [5]. BRCA1 has also been reported to be a substrate of Chk2 at S988 [122].

The BRCT domain of BRCA1 has been indicated important for cell cycle checkpoint, HR and tumor suppression [3,5,7-10]. The recent mouse model expressing a BRCT mutant of BRCA1 (S1598F, corresponding to human BRCA1 S1655F) incapable of binding to phospho-peptides in place of the wild type allele of BRCA1 failed to suppress breast tumor development further confirms that the BRCT domain and its capability to bind phosphorylated protein is required for the tumor suppressor function of BRCA1 [21]. Through its BRCT phospho-binding ability BRCA1 forms at least three mutually exclusive complexes by binding to phosphorylated proteins Abraxas, Bach1 and CTIP. The A, B and C complexes, at lease partially undertake BRCA1's role in mechanisms of cell cycle checkpoint and DNA repair that maintain genome stability (Figure 3), although it remains largely unknown how the formation of these complexes are regulated or coordinated. Many other proteins have also been reported binding to the BRCT domains of BRCA1 although it is not clear whether these proteins bind with a direct interaction [115,116]. It is likely additional BRCT associated complexes exist carrying additional roles of BRCA1 in a number of diverse cellular processes in maintaining genome stability and tumor suppression.

Although it is evident that BRCT domains are essential for BRCA1's tumor suppression, it remains unclear how the BRCT domain associated complexes are coordinated with signaling complexes that associate through other regions of the BRCA1 protein. Together, BRCA1 emerges to be a central mediator of the cellular mechanism that maintains genome stability that brings together multiple signaling complexes in response to DNA damage, yet much remains to be learned to fully appreciate the role of BRCA1 as a tumor suppressor.

## Competing interests

The authors declare that they have no competing interests.

## Authors' contributions

BW is the sole author of this manuscript.
